# Production of Cyanotoxins by *Microcystis aeruginosa* Mediates Interactions with the Mixotrophic Flagellate *Cryptomonas*

**DOI:** 10.3390/toxins11040223

**Published:** 2019-04-15

**Authors:** Sarah DeVaul Princiotta, Susan P. Hendricks, David S. White

**Affiliations:** Hancock Biological Station and Department of Biological Sciences, Murray State University, Murray, KY 42071, USA; shendricks@murraystate.edu (S.P.H.); dwhite@murraystate.edu (D.S.W.)

**Keywords:** microcystin, mixotrophy, phytoplankton, cyanobacterial blooms, *Microcystis*, trophic interactions

## Abstract

Eutrophication of inland waters is expected to increase the frequency and severity of harmful algal blooms (HABs). Toxin-production associated with HABs has negative effects on human health and aquatic ecosystem functioning. Despite evidence that flagellates can ingest toxin-producing cyanobacteria, interactions between members of the microbial loop are underestimated in our understanding of the food web and algal bloom dynamics. Physical and allelopathic interactions between a mixotrophic flagellate (*Cryptomonas* sp.) and two strains of a cyanobacteria (*Microcystis aeruginosa*) were investigated in a full-factorial experiment in culture. The maximum population growth rate of the mixotroph (0.25 day^−1^) occurred during incubation with filtrate from toxic *M. aeruginosa*. *Cryptomonas* was able to ingest toxic and non-toxic *M. aeruginosa* at maximal rates of 0.5 and 0.3 cells day^−1^, respectively. The results establish that although *Cryptomonas* does not derive benefits from co-incubation with *M. aeruginosa*, it may obtain nutritional supplement from filtrate. We also provide evidence of a reduction in cyanotoxin concentration (microcystin-LR) when toxic *M. aeruginosa* is incubated with the mixotroph. Our work has implications for “trophic upgrading” within the microbial food web, where cyanobacterivory by nanoflagellates may improve food quality for higher trophic levels and detoxify secondary compounds.

## 1. Introduction

Freshwater ecosystems world-wide face threats of deterioration through the proliferation of toxin-producing cyanobacteria [1,2]. Increased occurrence of cyanobacteria blooms is evident in eutrophic waters that receive significant nutrient inputs from surrounding watersheds [3,4]. Although it remains difficult to predict the causes of harmful algal bloom (HAB) formation, many studies point to nutrient concentration as a primary driving force in inland waters [5,6,7]. Analyses of the 2012 US Environmental Protection Agency National Lakes Assessment (EPA NLA) revealed that distribution of potentially toxigenic cyanobacterial taxa was related to regional water quality trends, particularly total phosphorus [8]. However, the importance of nitrogen as a predictor of cyanobacterial biomass cannot be disregarded [1]. Consequences of nutrient pollution are compounded by changes in global climate, as many species of cyanobacteria favor warm surface waters and sustained periods of stratification [9,10,11].

Cyanobacteria can produce a suite of compounds, many of which are toxic to aquatic biota and humans [12]. Loftin et al. [13] identified potential microcystin-producing taxa in 95% of samples taken from 1161 lakes and reservoirs across the contiguous United States. *Microcystis*, a potent microcystin-producer, was the most commonly detected cyanobacteria genus. Cyanobacteria blooms typically contain a mixed community of cyanobacteria that can include toxic and non-toxic strains of the same species [5]. It remains a challenge to predict triggers of toxin production across species and systems because production of cyanotoxins are not always associated with biovolume [14,15], although exceptions have been documented [16,17].

In addition to nutrient concentrations, multiple trophic interactions within the aquatic community govern the formation and degradation of cyanobacteria blooms, particularly interactions with zooplankton [18,19,20], filter-feeding fish [21], and mollusks (e.g., zebra mussels, [22]). Cyanobacteria can evade grazing through toxin production, colony formation, and reduced food quality [18,23,24]. Similar inducible defenses have been documented after exposure to protozoans [25,26]. Several studies have also demonstrated that heterotrophic protists can maintain high growth rates when grazing on toxic or non-toxic cyanobacteria [27,28,29]. In conjunction with heterotrophic forms, it has been suggested that phagotrophic phytoplankton that utilize mixed nutrition (mixotrophy) also can be effective grazers of cyanobacteria.

**Figure 1:**
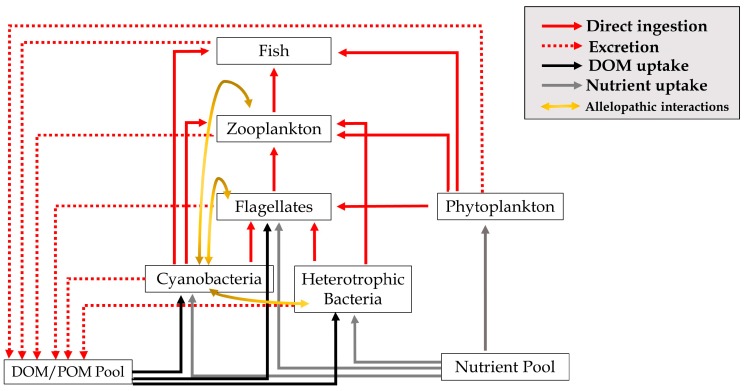
Conceptual diagram of an aquatic food web that includes and emphasizes the role of flagellates such as *Cryptomonas*. Flagellates are represented by autotrophic, heterotrophic, and mixotrophic forms. Phytoplankton refer to non-flagellated organisms. DOM = dissolved organic matter, POM = particulate organic matter.

Mixotrophy, a combination of photosynthesis with ingestion of particulate organic matter, has been recognized within a range of taxonomic groups [30]. Field studies reveal that mixotrophic protists are often numerically dominant in freshwater ecosystems and can exhibit a greater grazing impact on the bacterial community than heterotrophic forms [31,32,33]. Mixotrophic nutrition is remarkably plastic, allowing organisms to change their relative reliance on ingestion or photosynthesis based on environmental conditions or biotic pressures [34,35]. Consequently, mixotrophic protists are generally separated into categories along a nutritional gradient [36,37]. For example, species of *Dinobryon* (a genus of Chrysophyceae or golden algae) are facultative phagotrophs that supplement growth with particulate prey under oligotrophic conditions [35,38]. On the other hand, species of *Ochromonas* and *Poteriochromonas* (Chrysophyceae) exhibit a reduced dependence on photosynthesis except when prey concentration is limiting [39,40]. Several mixotrophic chrysophytes are able to feed directly on toxic, bloom-forming cyanobacteria [41,42,43,44]. Notably, species of mixotrophic flagellates that have been identified to ingest *Microcystis*, including *Ochromonas* and *Poteriochromonas,* are predominantly phagotrophic, with reduced contributions of phototrophy to overall metabolism [45,46]. Though work has been done to assess feeding by mixotrophs on *Microcystis*, many studies are limited to the heterotrophic-extreme of the nutritional gradient. Further, although it is well known that heterotrophic and mixotrophic protists take an intermediary role between zooplankton production and the microbial community, few studies have explored this trophic link (see Figure 1).

The purpose of this work was to examine biotic relationships between a mixotrophic flagellate (*Cryptomonas* sp., hereafter *Cryptomonas*) and two strains of bloom-forming cyanobacteria (toxic and non-toxic *Microcystis aeruginosa*, hereafter tox-*Microcystis* and nt-*Microcystis*). We also investigated the ability of *Cryptomonas* to ingest both *Microcystis* forms through the disappearance of fluorescently labeled prey. *Cryptomonas* and *Microcystis* were chosen because both are common in lakes and ponds at the same times of year and most likely have some competitive interactions in nature. The Cryptophyceae are a common group of photosynthetic flagellates that serve as a high-quality food source for crustacean grazers [47,48]. Reports of bacterivory by species of *Cryptomonas* are varied, but most studies suggest that the mixotroph is a facultative phagotroph that derives its nutrition primarily from photosynthesis [49,50]. Physical and allelopathic relationships were investigated under varying macronutrient conditions (nitrogen and phosphorus) in a full-factorial design with *Cryptomonas* and tox- or nt-*Microcystis* grown as a monoculture, in direct co-culture, and with the addition of reciprocal filtrate. The goals were to determine (1) if *Cryptomonas* is able to ingest and supplement growth with either tox- or nt-*Microcystis;* and (2) if production of microcystin-LR (hereafter MC-LR) by tox-*Microcystis* is influenced by the presence of *Cryptomonas*. Through work in culture, we demonstrate that *Cryptomonas* does not derive growth benefits from incubation with either strain of *Microcystis*, and maintains low grazing rates that vary with toxicity of prey and background nutrient concentrations. However, incubation with *Cryptomonas* or its filtrate led to a significant decline in population growth of tox-*Microcystis*.

## 2. Results

### 2.1. Biotic Interactions between Cryptomonas and nt-Microcystis

**Figure 2:**
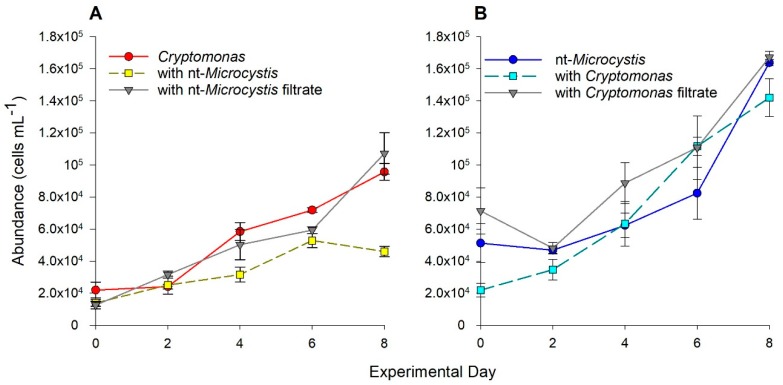
Changes in cell abundance of (**A**) *Cryptomonas* and (**B**) non-toxic*Microcystis* (nt-*Microcystis*) over the experimental period (eight days) when grown alone in monoculture, co-culture, or with reciprocal filtrate. Error bars represent standard error of the mean from three replicate samples.

Cell abundance of *Cryptomonas* increased during the experimental period (eight days) when grown alone, in the presence of, and with filtrate from, nt-*Microcystis* (Figure 2B). There was a significant effect of treatment (F_2,30_ = 22, *p* < 0.001), day (F_4,30_ = 73, *p* < 0.001) and interaction (F_8,30_ = 6, *p* < 0.001) on *Cryptomonas* cell abundance. After eight days of incubation alone or with filtrate from nt-*Microcystis*, the populations of *Cryptomonas* increased by 77% and 88%, reaching average final cell densities of 9.6 × 10^4^ and 1.1 × 10^5^ cells mL^−1^, respectively. However, in direct co-culture with nt-*Microcystis*, abundance of *Cryptomonas* increased by only 37%, reaching an average cell density of 4.6 × 10^4^ cells mL^−1^ (Figure 2B). Cell abundance of *Cryptomonas* on the final day of experimentation in the co-culture treatment was significantly lower than that when grown alone or with filtrate from nt-*Microcystis* (*p*_alone, together_ < 0.001, *p*_filt, together_ < 0.001).

Cell abundance of nt-*Microcystis* also increased when cultured with *Cryptomonas* (Figure 2A). There was a significant effect of treatment (F_2,30_ = 6.4, *p* = 0.005) and day (F_4,30_ = 64, *p* < 0.001) but no interactive effect (F_8,30_ = 1.7, *p* = 0.146) on nt-*Microcystis* cell abundance over the 8-day experimental period. On the final day of incubation, there was no significant difference in cell abundance of nt-*Microcystis* among treatments (*p*_filt, together_ = 0.21, *p*_filt, alone_ = 0.97, *p*_alone, together_ = 0.30), at an average of 1.6 × 10^5^ cells mL^−1^ (± 7.9 × 10^3^).

**Figure 3:**
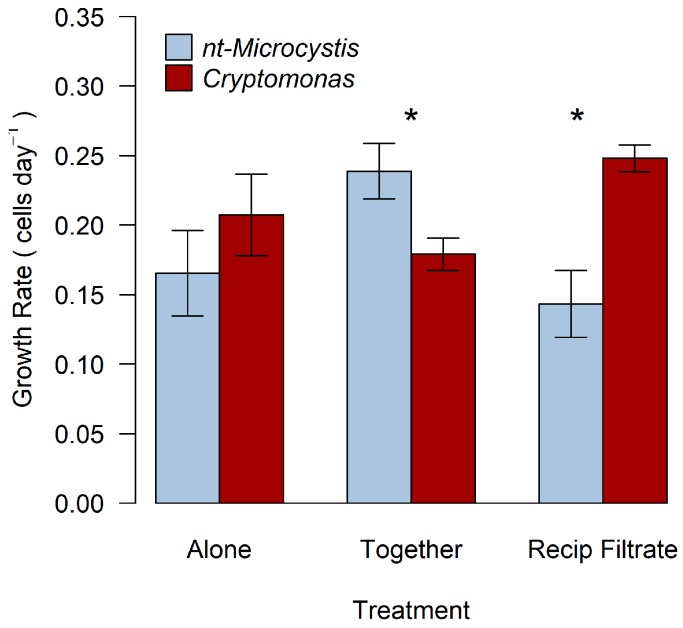
Population growth rate (cells day^−1^) of nt-*Microcystis* and *Cryptomonas* when grown alone in monoculture, incubated in co-culture, and with reciprocal filtrate. Growth rate was determined by slope of the linear regression of ln-transformed cell abundance values plotted over time (days). Asterisks indicate statistically significant differences between species within the same treatment (*p* < 0.05). Errors bars represent standard error of the mean from three replicate samples.

Population growth rate (cells day^−1^) of *Cryptomonas* over the eight-day period was 0.20 day^−1^, 0.18 day^−1^, and 0.25 day^−1^ when grown alone, in co-culture with nt-*Microcystis*, and with reciprocal filtrate, respectively (Figure 3). Growth rate in co-culture or with filtrate from nt-*Microcystis* was not different from when *Crytomonas* was grown alone (*p*_together, alone_ = 0.53, *p*_filt, alone_ = 0.29). However, population growth rate of the mixotroph was significantly higher when cultured with filtrate from nt-*Microcystis* than in direct, co-culture with the cyanobacteria (*p*_together, filt_ = 0.05). Population growth rate (cells day^−1^) of nt-*Microcystis* grown alone, in co-culture with *Cryptomonas*, and in the presence of reciprocal filtrate was 0.18 day^−1^, 0.25 day^−1^, and 0.19 day^−1^, respectively (Figure 3). nt-*Microcystis* exhibited the highest population growth rate in the physical presence of *Cryptomonas* (p_together, alone_ = 0.04, *p*_filt, together_ = 0.09, *p*_filt, alone_ = 0.86). The population growth rates of nt-*Microcystis* and *Cryptomonas* were not significantly different from one another when either were grown in monoculture (*p* = 0.28). However, nt-*Microcystis* exhibited a significantly higher population growth rate than *Cryptomonas* in the co-culture treatment (*p* = 0.02).

### 2.2. Biotic Interactions between Cryptomonas and tox-Microcystis

**Figure 4:**
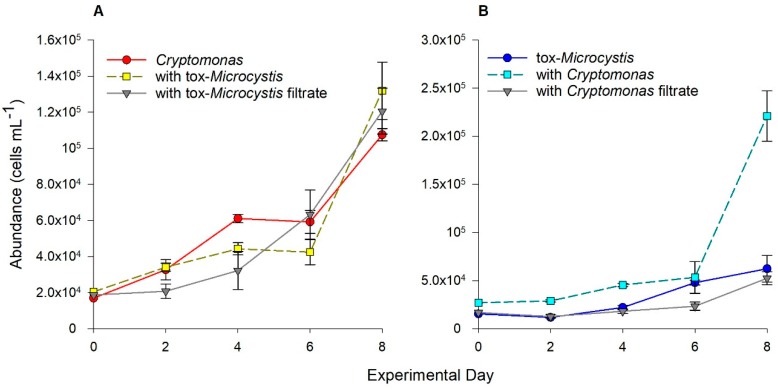
Changes in cell abundance of (**A**) *Cryptomonas* and (**B**) toxic-*Microcystis* (tox-*Microcystis*) over the experimental period (8 days) when grown alone in monoculture, co-culture, or with reciprocal filtrate. Error bars represent standard error of the mean between three replicate samples. Note the change in scale between panels.

*Cryptomonas* exhibited positive trends in cell abundance within all treatment combinations with tox-*Microcystis.* When grown alone, in co-culture, or with filtrate from tox-*Microcystis*, *Cryptomonas* reached a final cell abundance of 1.2 × 10^5^ cells mL^−1^ (± 7.1 × 10^3^), increasing by 85% (Figure 4B). There was a significant effect of day (ANOVA, F_4,30_ = 80.6, *p* < 0.001), but not treatment, on cell abundance of *Cryptomonas* over the experimental period. On the final day of incubation, cell abundance of *Cryptomonas* was not significantly different among treatments (*p*_filt, together_ = 0.55, *p*_filt, alone_ = 0.46, *p*_alone, together_ = 0.08).

At the end of the incubation period, cell abundance of tox-*Microcystis* was significantly higher when grown in co-culture with *Cryptomonas* (*p*_together, filt_ = 0.001, *p*_together, alone_ = 0.001), reaching 2.2 × 10^5^ cells mL^−1^ (Figure 4A). There was a significant effect of day (ANOVA, F_4,30_ = 55, *p* < 0.001), treatment (ANOVA, F_2,30_ = 44, *p* < 0.001), and interaction (ANOVA, F_8,30_ = 18, *p* < 0.001) on cell abundance of tox-*Microcystis*. In treatments with *Cryptomonas* filtrate or in monoculture, tox-*Microcystis* reached maximum abundances of 5.2 × 10^4^ cells mL^−1^ and 6.2 × 10^4^ cells mL^−1^, respectively (*p* = 0.73). However, a final cell abundance of 2.21 × 10^5^ cells mL^−1^ was reached after direct co-culture with *Cryptomonas* (Figure 4A).

**Figure 5:**
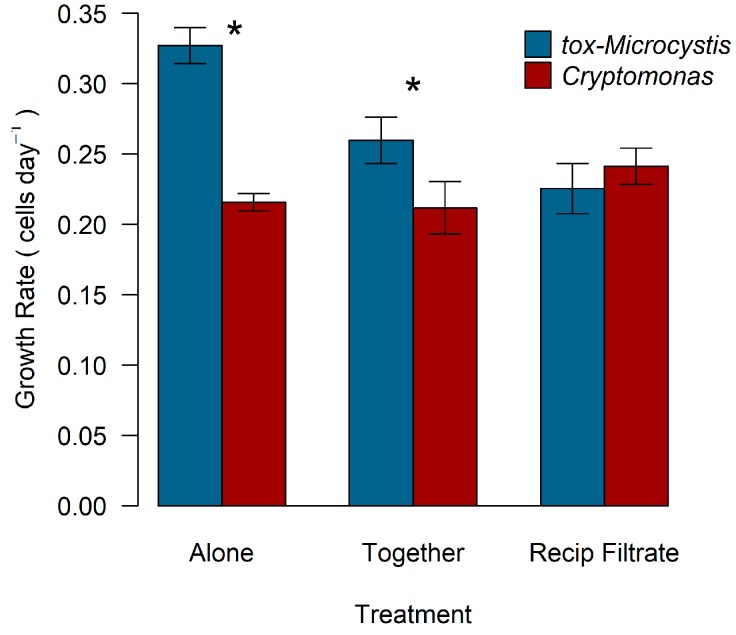
Population growth rate (cells day^−1^) of tox-*Microcystis* and *Cryptomonas* when grown alone in monoculture, incubated in co-culture, and with reciprocal filtrate. Growth rate determined by slope of the linear regression of ln-transformed cell abundance values plotted over time (days). Asterisks indicate statistically significant differences between species within the same treatment (*p* < 0.05). Errors bars represent standard error of the mean from three replicate samples.

Population growth rate (cells day^−1^) of *Cryptomonas* was 0.22 day^−1^, 0.21 day^−1^, and 0.24 day^−1^ when grown alone, in co-culture with tox-*Microcystis*, and with reciprocal filtrate, respectively (Figure 5). Population growth rate of *Cryptomonas* in monoculture did not differ between experiments with varying macronutrient concentration, despite a reduction in nitrogen and phosphorus in the second series (*t*-test, *t* = 0.36, *p* = 0.75). The toxic strain of *Microcystis* exhibited population growth rates of 0.33 day^−1^, 0.26 day^−1^, and 0.23 day^−1^ when grown alone, in co-culture with *Cryptomonas*, and with reciprocal filtrate, respectively (Figure 5). Relative to population growth rate alone in monoculture, tox-*Microcystis* exhibited significantly lower rates in co-culture (*p*_palone, together_ = 0.02) or with reciprocal filtrate from *Cryptomonas* (*p*_palone, filt_ = 0.001). Toxic-*Microcystis* exhibited a higher population growth rate than *Cryptomonas* when both were grown alone in monoculture (*p* = 0.001) and in direct co-culture (*p* = 0.04).

### 2.3. Toxicity of Microcystis in Response to Cryptomonas

Intracellular and dissolved fractions of nt-*Microcystis* did not contain detectable microcystin-LR (MC-LR). The toxic strain of *Microcystis* was confirmed to contain intracellular MC-LR values of 0.06 pg cell^−1^ or 347 ng mL^−1^ during the exponential phase of growth alone in nutrient enriched media. The cyanotoxin was not detectable in the dissolved fraction of tox-*Microcystis* in unialgal culture nor experimental treatments. This was expected because microcystins are generally contained intracellularly until cell lysis [12]. Description of MC-LR results will hereafter refer to intracellular toxin concentrations.

**Figure 6:**
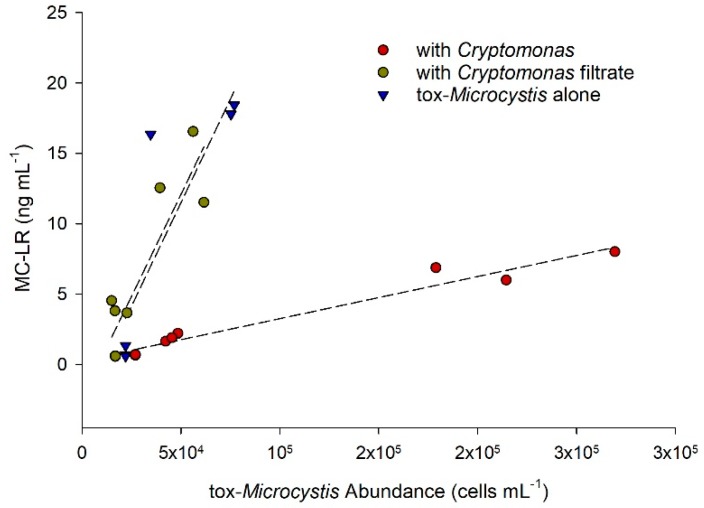
Relationship between total microcystin-LR (MC-LR, L=leucine, R=arginine, located at positions 2 and 4, respectively, on cyclic molecule) concentration and cell abundance of tox-*Microcystis* during incubation with *Cryptomonas*, filtrate from the mixotroph, or alone in monoculture. Data includes three replicate values from each day of experimentation.

**Figure 7:**
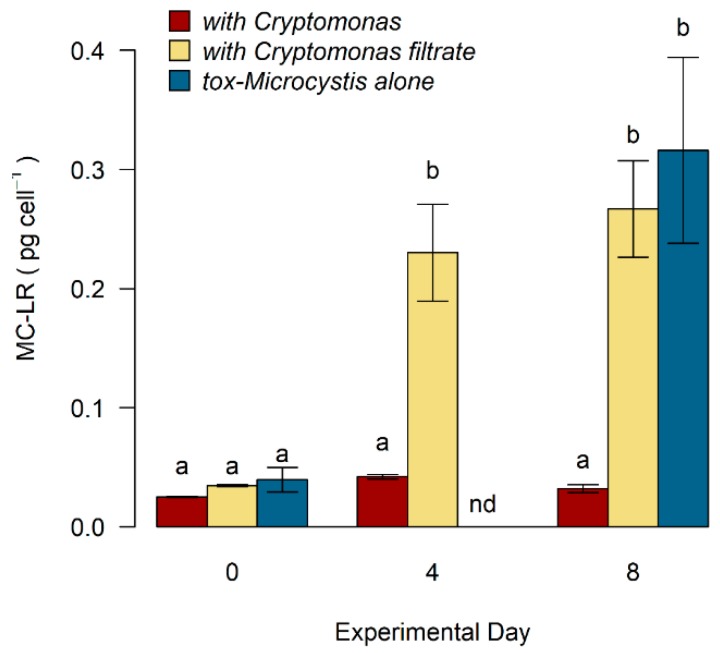
Intracellular concentration of microcystin-LR (MC-LR) in tox-*Microcystis* after incubation with *Cryptomonas*, filtrate from the mixotroph, or alone in monoculture. Letters above bars indicate results of non-parametric ANOVA (Kruskal–Wallis) performed on MC-LR cell^−1^ within each treatment day. Note that MC-LR was not measured in tox-*Microcystis* monoculture on day 4 (nd = no data).

Overall, MC-LR concentration was linearly related to tox-*Microcystis* abundance within each treatment (Figure 6). There was a significant increase in concentration of MC-LR (cell^−1^) over time when tox-*Microcystis* was grown in monoculture, reaching a maximum of 0.32 pg cell^−1^ on day eight (Table 1, Figure 7). However, when tox-*Microcystis* was grown in co-culture with *Cryptomonas*, a peak of only 0.04 pg cell^−1^ MC-LR was reached on the fourth day of experimentation. In the presence of filtrate derived from *Cryptomonas*, MC-LR cell^−1^ in tox-*Microcystis* also increased over the eight-day experimental period, reaching a maximum of 0.27 pg cell^−1^ on the final day.

On the initial day of incubation (day 0), there was no significant difference in intracellular MC-LR cell^−1^ between the treatments (0.03 pg cell^−1^ ± 0.004, Kruskal–Wallis *p* = 0.06, Figure 7). After eight days, MC-LR cell^−1^ was approximately 89% lower in treatments where tox-*Microcystis* was grown in the presence of *Cryptomonas* compared with cells grown alone in monoculture or with filtrate from the mixotroph (*p*_alone, together_ = 0.02, *p*_alone, filt_ = 0.78, *p*_filt, together_ = 0.04).

### 2.4. Ability of Mixotrophic Cryptomonas to Ingest tox- and nt-Microcystis

Measurements of MC-LR within the intracellular portion of fluorescently labeled tox-*Microcystis* (tox-FLM) were below the detection limit. Values of 4.62 ng mL^−1^ were measured in the dissolved portion of tox-FLM, indicating that preparation of labeled cells may have caused the cyanobacteria to release intracellular toxins. Upon view via microscopy, cellular membranes appeared intact. It was assumed that *Cryptomonas* was exposed to MC-LR during incubation with tox-FLM because both filtrate and whole cells were provided.

**Figure 8:**
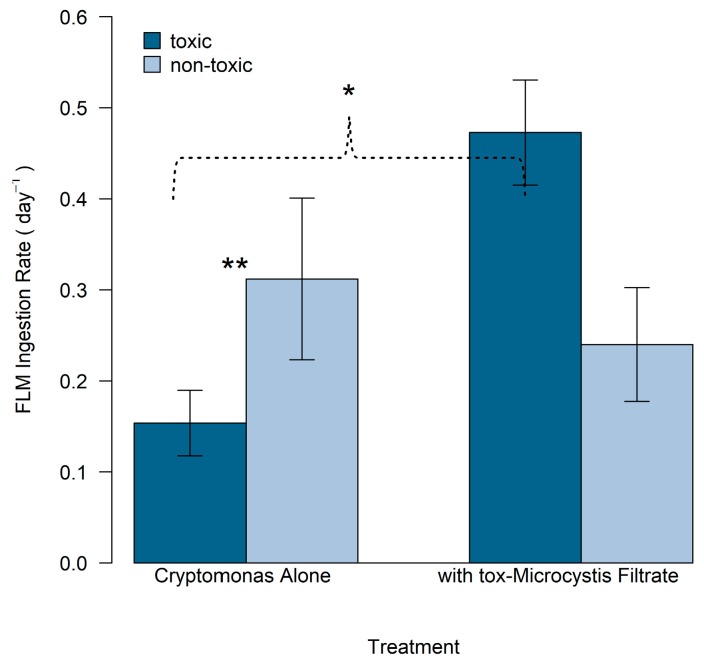
Ingestion rate (cells day^−1^) of toxic and non-toxic fluorescently labeled *Microcystis* (as measured by disappearance) in treatments with either *Cryptomonas* alone or cultured with filtrate from tox-*Microcystis*. Error bars represent standard error of the mean from three replicate samples. Asterisks indicate statistically significant differences (*p* < 0.05): * Between ingestion rate of toxic FLM when *Cryptomonas was* grown alone or with addition of tox-*Microcystis* filtrate; ** Between ingestion rate of toxic and non-toxic FLM when *Cryptomonas* was grown alone.

There was no evidence of uptake of non-toxic fluorescently labeled *Microcystis* (nt-FLM) by *Cryptomonas* over a period of two hours after incubation in monoculture, in co-culture with nt-*Microcystis*, nor with filtrate from the cyanobacteria. However, abundance of tox-FLM and nt-FLM declined significantly after incubation with *Cryptomonas* that was grown either alone in monoculture or incubated with filtrate from tox-*Microcystis* (Figure 8, Table 2). When incubated alone in monoculture, ingestion rates of tox-FLM and nt-FLM by *Cryptomonas* were 0.15 and 0.31 cells day^−1^, respectively (Figure 8). Therefore, the ingestion rate was greater on nt-*Microcystis*. Incubation of *Cryptomonas* with filtrate from tox-*Microcystis* led to a significant increase in ingestion of tox-FLM (0.5 cell day^−1^, *p* < 0.0001), but did not influence ingestion of nt-FLM (*p* = 0.19).

## 3. Discussion

In all the mixed-culture experiments, the physical effects of both tox- and nt-*Microcystis* on the population growth rate of *Cryptomonas* were insignificant, indicating that growth of the mixotroph was not enhanced by the presence of a potential cyanobacterial food source. Although the population growth rate of *Cryptomonas* was reduced in the co-culture with both strains of *Microcystis*, it was not significantly different relative to that alone. In each set of experiments, the highest growth rate of *Cryptomonas* occurred in the presence of cyanobacterial filtrate, suggesting that allelopathic compounds from *Microcystis* provide nutritional value to the mixotroph. Facilitative effects of toxic cyanobacterial filtrate, especially at low concentrations, have been shown to support growth of *Cyptomonas ovata* [51]. Alternatively, filtrate from *Microcystis* may have fueled growth of heterotrophic bacteria that can be grazed by *Cryptomonas*. Studies in natural systems have identified members of the Cryptophyta can be responsible for 70% of total bacterivory [52].

Interactions between cyanobacteria and heterotrophic bacteria often have been described in context of the phycosphere, the nutrient-rich microenvironment on the periphery of cyanobacterial cells [53,54]. Cyanobacterial filtrate has been shown to support bacterial growth and production [55,56]. For example, Shen et al. [57] showed significantly higher population growth of heterotrophic bacteria when grown in the presence of *Microcystis aeruginosa*. Both quantity and quality of filtrate, as well as the physiological state of the cyanobacterial cells, are expected to influence associated bacteria. Though we did not fully characterize the filtrate from both strains of *Microcystis*, there were no significant differences in dissolved organic carbon (DOC) concentration between filtrates (Table S1). Further, filtrate derived from tox-*Microcystis* did not contain detectable MC-LR. Therefore, DOC nor absence of cyanotoxin does not likely drive the advantages afforded by exposure to nt-*Microcystis* filtrate.

Although it is difficult to disentangle the effects of nutrient availability and toxicity, our results suggest that the physical presence of *Cryptomonas* had contrasting impacts on growth of two strains of *Microcystis* that varied in toxicity. Whereas the population growth rate of nt-*Microcystis* was greatest in co-culture with *Cryptomonas*, that of tox-*Microcystis* was significantly reduced in the physical presence of the mixotroph (relative to monoculture). There was also a reduction in the growth rate of tox-*Microcystis* when incubated with filtrate from *Cryptomonas*, suggesting a negative effect of compounds released by the mixotroph. Therefore, the negative effect of *Cryptomonas* on tox-*Microcystis*, at least under low nutrient conditions, is likely through a combination of grazing and allelopathy. This contradicts work by Wilken et al. [44], where *Ochromonas* filtrate did not influence abundance of neither toxic nor non-toxic *M. aeruginosa*. Experimental differences between our work and others allude to the role of the mixotrophic gradient in predicting interactions between flagellates and cyanobacteria.

We expected that the population growth of nt-*Microcystis* would be reduced in the presence of *Cryptomonas*, either by competition or direct ingestion. However, population growth of nt-*Microcystis* was not inhibited by *Cryptomonas*, and the overall growth rate was highest in the co-culture with the mixotroph. In fact, nt-*Microcystis* reached approximately the same final cell abundance in all experimental treatments. Our results suggest that the competitive interactions are not strong enough for *Cryptomonas* to provide top-down control of nt-*Microcystis*, particularly under high-nutrient conditions used in the experimentation. It is well known that *Microcystis* is a superior competitor under high nutrient conditions, such as those provided by nutrient-replete media in the first series of experiments [5,58]. The mixotrophic Chrysophyte, *Ochromonas*, was able to suppress growth of toxic *Microcystis aeruginosa*, but only under a reduction in nitrogen concentration (20 μmol L^−1^ NH_4_CL) [39]. In our work, *Cryptomonas* was also able to suppress growth rate of tox-*Microcystis* under nutrient-deplete conditions. Both studies suggest that mixotrophic flagellates may be effective biological control agents only in oligotrophic systems where cyanobacterial blooms are less pervasive.

Ingestion rates of tox- and nt-*Microcystis* by *Cryptomonas* were lower than reported for other mixotrophs. *Ochromonas*, a mixotroph on the heterotrophic end of the nutritional gradient, has been documented to maintain high ingestion rates on toxic strains of *Microcystis* [42,44]. For example, *Ochromonas* exhibited a maximum ingestion rate of 5.7 prey^−1^ day^−1^ and 2.1 prey^−1^ day^−1^ on toxic and non-toxic strains of *M. aeruginosa*, respectively [44]. Flagellate grazing in the previously mentioned study was conducted with live cyanobacterial cells, whereas our work was done with heat-killed, fluorescently labeled prey. Comparisons of the ingestion rates between this and other studies must be interpreted cautiously, as the use of fluorescently labeled tracers carries inherent bias. For example, studies in a heterotrophic flagellate revealed reduced feeding on DTAF-labeled bacteria, likely due to alterations to cell–surface properties [59].

The use of fluorescent prey was chosen to separate the effects of competition and grazing. Differences in ingestion rates between this and other studies may have resulted from initial inoculation density of FLM, where during our work FLM were added at a lower, yet still ecologically relevant, concentration. Other work on ingestion of cyanobacteria by mixotrophic flagellates suggests an increase in flagellate growth and ingestion rate with cyanobacterial density up to 10^6^ cells mL^−1^ [41,44]. Grazing rate depends not only on the abundance of prey, but also population density of the predator. For example, ingestion rate of *Ochromonas* was reduced at concentrations greater than 10^5^ cells mL^−1^, when intraspecific interference was hypothesized to reduce grazing on toxic *M. aeruginosa* [39]. However, no study has examined feeding on toxic or non-toxic *Microcystis* by *Cryptomonas,* and feeding rates are likely variable among flagellates. It should be noted that the cyanobacteria strains studied here are not isogenic pairs and observed differences may have resulted from attributes other than the ability to produce MC-LR. However, many studies emphasize prey size and shape as factors that greatly influence grazing [60], and such traits were similar between strains.

During incubation in monoculture under low-nutrient conditions, *Cryptomonas* exhibited a greater ingestion rate on nt-FLM. However, incubation with filtrate from tox-*Microcystis*, which did not contain MC-LR, led to a significant increase in ingestion of tox-FLM. Although tox-FLM did not contain detectable intracellular MC-LR, the surrounding filtrate contained levels above our limit of detection. Therefore, cyanobacterivory by *Cryptomonas* was not inhibited by presence of the cyanotoxin MC-LR. Other work using laboratory cultures has documented the ability of mixotrophic and heterotrophic flagellates to tolerate microcystins [42]. We suggest that filtrate derived from the toxic strain may have provided a source of nutritional supplement that supported increased grazing by *Cryptomonas*. Addition of cyanobacterial filtrate may have induced a shift in nutritional mode from photosynthesis to heterotrophy by supporting photosynthetic machinery. Alternatively, exposure to MC-LR may have inhibited the photosynthetic machinery of *Cryptomonas* and constrained the mixotroph to utilize cyanobacterivory.

Concentrations of intracellular MC-LR were significantly reduced in tox-*Microcystis* after co-incubation with *Cryptomonas*. A similar result has been observed for *Ochromonas* and *Poterioochromonas* [43,61]. Our results imply that *Cryptomonas* has a negative impact on production or accumulation of MC-LR. We propose that the mixotrophic flagellate, *Cryptomonas*, may be able to biodegrade MC-LR though ingestion of toxin-producing *Microcystis*. Ingestion of microcystin-producing cyanobacteria by mixotrophic flagellates may act to supply N-rich compounds. Further work is required to substantiate the biodegradation theory, as we did not discriminate between microcystins within mixotrophic or cyanobacterial cells. However, a similar study with *Poterioochromonas malhamensis* indicated that MC-LR does not accumulate within the cell of the predatory mixotroph [61]. Cyanotoxin concentrations were corrected to cellular abundance of *Microcystis*, therefore results are not likely due to a depletion in the cyanobacteria population. However, similar trends in MC-LR mL^−1^ over the experimental period were observed without correction for cell abundance (Figure S1). Further, tox-*Microcystis* reached the greatest cell abundance on the final day of incubation with *Cryptomonas*, during which there was a significant reduction in concentration of MC-LR.

Competition, predation, and allelopathic interactions between cyanobacteria and mixotrophic flagellates are an underrepresented trophic link that may be important in structuring the microbial food web (see Figure 1). Metabolic coupling between cyanobacteria and mixotrophic flagellates may depend on their position on the nutritional gradient. Laboratory work in this and other studies support the theory that the presence of mixotrophic protists can have an impact on cyanobacteria, including reductions in population growth rate and production of cyanotoxins. Whereas mixotrophs on the heterotrophic extreme of the nutritional gradient seem to gain growth benefits from cyanobacterivory, population growth of *Cryptomonas*, a primary phototroph, was largely unaffected by the presence of toxic and non-toxic strains of *Microcystis*. Instead, *Cryptomonas* may use organic carbon supplied from cyanobacterial filtrate to maintain, but not enhance, its growth. The microbial loop has revealed that bacteria are a critical link between dissolved organic matter and higher trophic levels through ingestion by heterotrophic and mixotrophic flagellates. Cyanobacterivorous protists may also mediate nutrient remineralization though detoxification of secondary metabolites and provide a subsidy to the classic food web.

## 4. Materials and Methods

Cultures of *Cryptomonas* sp. (CPCC #336) and *Microcystis aeruginosa* were purchased from the Canadian Phycological Culture Centre (CPCC) at the University of Waterloo, Ontario, Canada. *Microcystis aeruginosa* (CPCC #300, referred to as tox-*Microcystis*) was indicated to produce microcystins up to 200 µg g^−1^ of dry weight. The non-toxic strain of *M. aeruginosa* (CPCC #632, nt-*Microcystis*) originated from the University of Texas Culture Collection at University of Texas at Austin (UTEX LB 2061) and initially was isolated from Lake Mendota in Wisconsin, USA. Cultures were unialgal but contained heterotrophic bacteria. All cultures were maintained in an enriched Bold’s Basal Medium (BBM) at 20 °C. Light was provided by cool-white fluorescent bulbs under a 12:12 light:dark cycle of 100 µmol photons m^−2^ s^−1^ in a Percival Intellus environmental controller (Percival Scientific, Perry, IA, USA).

### 4.1. Preparation and Analysis of Algal Filtrate

Filtrate was prepared by passing unialgal culture through a sterile 0.2-µm filter unit (Nalgene, Nalge Nunc International Corp., Rochester, NY, USA). Filter size was chosen as to reduce the addition of heterotrophic bacteria from non-axenic cultures. Dissolved organic carbon was measured in replicate samples of filtrate from *Cryptomonas* and both tox- and nt-*Microcystis* via the heated persulfate wet oxidation technique on an Aurora 1030 TOC Analyzer with model 1088 Autosampler (OI Analytical, College Station, TX, USA).

### 4.2. Biotic Interactions between a Mixotrophic Flagellate, Cryptomonas, and Two Strains of Microcystis aeruginosa

Aliquots of each species were allowed to acclimate over 10 days to a temperature of 22 °C under a 12:12 light:dark cycle of 30 µmol photons m^−2^ s^−1^. Both were grown and maintained in highly enriched BBM (38 mg L^−1^ N, 63 mg L^−1^ P). After acclimation, *Cryptomonas* and nt-*Microcystis* were incubated in triplicate 250-mL culture flasks according to the following treatments: individual species as a monoculture, mixed culture of both species, and each species grown with reciprocal filtrate. Each treatment was inoculated according to the ratio of algae: media recommended by the CPCC; 5:1 for *Microcystis* and 15:1 for *Cryptomonas*. Mean final concentration of inoculate was 3.2 × 10^4^ cells mL^−1^. Treatments designated for addition of reciprocal filtrate received the same volume of filtrate represented by culture that was added to those in mixed treatments. Experimental manipulations between *Cryptomonas* and nt-*Microcystis* were maintained for 8 days after initial inoculation, with daily rotation, sub-sampling for cell abundance, and replenishment with an equal volume of BBM. Replicates with reciprocal filtrate were replenished with BBM or fresh filtrate on alternate days. Sub-samples for enumeration of cell abundance were fixed with 50% ice-cold glutaraldehyde and stored cold until analysis (<1 week).

Cultures used in the second series of experiments between *Cryptomonas* and tox-*Microcystis* were maintained under similar light and temperature conditions as described. During the acclimation period, aliquots of *Cryptomonas* and tox-*Microcystis* were serially transferred to a nutrient-deplete BBM with 3.3 mg L^−1^ TN and 0.3 mg L^−1^ TP. After acclimation, the experiment progressed as previously described with an 8-day incubation of daily rotation and media or filtrate replenishment. Sub-sampling for cell enumeration occurred on alternate days.

### 4.3. Cyanobacterivory by Cryptomonas

Ability of *Cryptomonas* to ingest strains of *Microcystis* (i.e., cyanobacterivory) was assessed by either uptake or disappearance of heat-killed, fluorescently labeled *Microcystis* (FLM) prepared from culture stocks). Toxic and non-toxic *Microcystis* were labeled with the fluorescent protein stain 5-(4,6-dichlorotriazin-2-yl) amino fluorescein (DTAF, λ_ex_ 485 nm, λ_em_ 516 nm) according to References [62,63]. Monocultures in exponential growth were concentrated via centrifugation at low speeds to avoid cell lysis. Pelletized culture was then resuspended in phosphate-buffered saline (pH 9) and incubated for 2 h at 70 °C with DTAF at 0.2 mg mL^−1^. After incubation, culture was concentrated by centrifugation, resuspended in 10 mL PBS, and unbound-DTAF was removed with three additional concentration-rinse cycles. Heat-killed, fluorescently labeled preparations (hereafter tox-FLM and nt-FLM), were stored at 5 °C in darkness prior to use. Sonification was used prior to addition to grazing experiments to reduce clumping.

On the final day of experimentation (day eight), treatments of *Cryptomonas* alone, *Cryptomonas* with nt-*Microcystis*, and *Cryptomonas* with filtrate from nt-*Microcystis* received an addition of nt-FLM at tracer levels, equivalent to 7 × 10^4^ cells ml^−1^. Sub-samples were taken immediately after nt-FLM addition to account for background coincidence and again after 15, 30, 60, and 120 min. Samples were fixed with 2% ice-cold glutaraldehyde and stored in darkness prior to analysis.

Ability of *Cryptomonas* to ingest *Microcystis* also was evaluated through disappearance of tox- and nt-FLM. On the final day the second series of culture experiments (day eight), tox- and nt-FLM were independently added to treatments of *Cryptomonas* alone and that grown with filtrate from tox-*Microcystis*. Inoculation density was on the order of 5 × 10^4^ FLM mL^−1^. Replicates were incubated under the same experimental conditions, with subsamples taken immediately after tox- and nt-FLM addition and again after 24, 48, and 72 h. As a control, tox- and nt-FLM were incubated alone under the same conditions in tissue culture flasks. Samples were fixed with 2% ice-cold glutaraldehyde and stored in darkness.

### 4.4. Microcystin Analysis and Extraction

Intracellular and dissolved concentrations of microcystin (MC-LR) were determined by liquid chromatography/tandem mass spectrometry (LC-MS/MS) following modifications of the EPA Method 544 [64]. Intracellular MC-LR was measured by passing a 10-mL aliquot from each replicate through a 0.45-µm polycarbonate membrane (25 mm in diameter, Whatman, Maidstone, UK), which was placed in a scintillation vial with 2 mL 80% methanol and immediately frozen. After 16 h, the filter was gently swirled, and liquid was transferred to an additional sterile scintillation vial. The filter was rinsed three times with 80% methanol, for a final volume of 10 mL. Dissolved, extracellular MC-LR was determined from filtrate of original aliquot, which was stored at 5 °C until analysis (no more than 14 days). The same method was used to verify ambient concentration of intracellular and dissolved MC-LR in monocultures of both *Microcystis* strains grown at high cell densities and in tox-FLM used in cyanobacterivory experiments. Samples for MC-LR analysis during experimentation were taken on days 0 and 8 in tox-*Microcystis* grown alone, co-cultured with *Cryptomonas*, and grown with reciprocal filtrate. The MC-LR was also quantified on day four in the co-culture and reciprocal filtrate treatments.

Samples for intracellular and dissolved microcystin were prepared by solid-phase extraction by passing through a syringe-barrel cartridge with universal polymeric reversed phase sorbent (Oasis HLB 6cc, Waters Corp., Milford, MA, USA) on a vacuum manifold. Analytes were eluted from the column with a solution of methanol containing 5% distilled water with formic acid. Extracts were concentrated by evaporation with nitrogen gas and adjusted to a final volume of 0.25 mL^−1^ with methanol. The LC-MS/MS system consisted of dual Varian ProStar (210) pumps interfaced to a Varian 1200L triple-quadrupole mass spectrophotometer (Varian, Inc., Palo Alto, CA, USA). A standard curve was prepared from serial dilution of MC-LR stock in methanol (Beagle Bioproducts Inc., Columbus, OH, USA). Recovery of microcystin through the sample matrix as determined by comparison of extracted and non-extracted standard was 91%. The MC-LR concentration was standardized to cell abundance of *Microcystis* for analysis.

### 4.5. Cell Enumeration and Examination of Bacterivory by Fluorescence Microscopy

Sub-samples were prepared for cell enumeration by filtration of 200 µL onto a 25 mm, 0.2 µm white polycarbonate membrane (Millipore Sigma, Burlington, MA, USA). Filters were mounted onto slides with Vectashield mounting media (Vector Laboratories, Burlingame, CA, USA) containing DAPI stain (4’,6-diamidino-2-phenylindole) and enumerated using fluorescence microscopy with a Zeiss Axioplan at 400× magnification. *Microcystis* and *Cryptomonas* were first visualized by chlorophyll autofluorescence and verified by identification of nucleic material under UV excitation. Tox- and nt-FLM were enumerated under blue-light excitation.

### 4.6. Determination of Population Growth Rate and Ingestion Rate

All statistical analyses were performed using SigmaPlot (Systat Software, San Jose, CA, USA) and R software package “multcomp” [65]. Population growth rates (cells day^−1^) for each species were calculated by determining the slope of the linear regression of ln-transformed cell abundance values plotted over time. Growth rates of species across treatments were compared by two-way ANOVA, followed by post-hoc comparisons (Tukey honest significant difference test). Normality and equal variance were verified with Shapiro–Wilk and Brown–Forsythe tests, respectively. Due to the slight increase in inoculation density of nt-*Microcystis* alone or with filtrate from *Cryptomonas* (Figure 2B), growth rates of nt-*Microcystis* were also recalculated by considering the starting density as the mean cell abundance between days 0 and 2. In each treatment, the recalculated growth rate was not different than the original calculations. Therefore, original growth rate calculations were used for subsequent statistical analyses.

Rate of FLM disappearance, and therefore flagellate grazing rate, was determined by the slope of a linear regression through log-transformed FLM abundance over time. Comparison of rates of FLM disappearance in different treatments were performed with one-way ANCOVA after verification of normality (Shapiro–Wilk) equal variance (Levene), and equal slopes. Post-hoc pairwise comparisons were made by the Holm–Sidak method. One-way ANOVA and subsequent Tukey tests were used to compare intracellular MC-LR over treatments and days. In the event that assumptions of normality were not met (*n* = 1), Kruskal–Wallis one-way analysis of variance on ranks was used.

## Figures and Tables

**Table 1:** Results of one-way analysis of variance of intracellular MC-LR concentration (MC-LR cell^−1^) in tox-*Microcystis* either grown alone, with *Cryptomonas*, or with addition of filtrate derived from *Cryptomonas*. Asterisk indicates failure of normality test and subsequent use of ANOVA on ranks.

**Treatment**	**Variable**	**df**	**F**	***p***
*Microcystis* alone	day	1	12.309	0.025
*Microcystis* + *Cryptomonas*	day	2	14.888	0.005
*Microcystis* + *Cryptomonas*_filt_	day	2	14.222	0.005
**Day of Experiment**		**df**	**F**	***p***
0		2	5.600 *	0.061
4		1	21.400	0.010
8		2	8.919	0.016

**Table 2:** Results of one-way analysis of covariance (ANCOVA) for the equal slopes model of ingestion of fluorescently labeled *Microcystis* (FLM) by *Cryptomonas* with pairwise multiple comparisons (Holm–Sidak method). Tox = toxic FLM, nt = non-toxic FLM. Filtrate refers to tox-FLM filtrate.

**Variable**	**df**	**F**	***p***
Treatment	3	19.350	<0.001
Time	1	62.539	<0.001
Residual	31	-	-
**Treatment**	**FLM Type**		***p***
*Cryptomonas* alone	tox-nt		<0.001
*Cryptomonas* + filtrate	tox-nt		0.240
alone-filtrate	Tox		<0.001
alone-filtrate	nt		0.190

## Supplementary Materials

The following are available online at https://www.mdpi.com/2072-6651/11/4/223/s1, Figure S1: Concentration of microcystin-LR (MC-LR) without correction for cell abundance, as measured in tox-*Microcystis* after incubation with *Cryptomonas*, filtrate from the mixotroph, or alone in monoculture, Table S1: Average dissolved organic carbon (DOC) derived from unialgal cultures, measured in triplicate samples.

## References

[1] Beaulieu M., Pick F., Gregory-Eaves I. (2013). Nutrients and water temperature are significant predictors of cyanobacterial biomass in a 1147 lakes data set. Limnol. Oceanogr..

[2] Taranu Z.E., Gregory-Eaves I., Leavitt P.R., Bunting L., Buchaca T., Catalan J., Domaizon I., Guilizzoni P., Lami A., McGowan S. (2015). Acceleration of cyanobacterial dominance in north temperate-subarctic lakes during the Anthropocene. Ecol. Lett..

[3] Beaver J.R., Manis E.E., Loftin K.A., Graham J.L., Pollard A.I., Mitchell R.M. (2014). Land use patterns, ecoregion, and microcystin relationships in US lakes and reservoirs: A preliminary evaluation. Harmful Algae.

[4] Paerl H.W. (2018). Mitigating Toxic Planktonic Cyanobacterial Blooms in Aquatic Ecosystems Facing Increasing Anthropogenic and Climatic Pressures. Toxins.

[5] Dolman A.M., Rucker J., Pick F.R., Fastner J., Rohrlack T., Mischke U., Wiedner C. (2012). Cyanobacteria and cyanotoxins: The influence of nitrogen versus phosphorus. PLoS ONE.

[6] Rigosi A., Carey C.C., Ibelings B.W., Brookes J.D. (2014). The interaction between climate warming and eutrophication to promote cyanobacteria is dependent on trophic state and varies among taxa. Limnol. Oceanogr..

[7] Marmen S., Aharonovich D., Grossowicz M., Blank L., Yacobi Y.Z., Sher D.J. (2016). Distribution and Habitat Specificity of Potentially-Toxic Microcystis across Climate, Land, and Water Use Gradients. Front. Microbiol..

[8] Beaver J.R., Tausz C.E., Scotese K.C., Pollard A.I., Mitchell R.M. (2018). Environmental factors influencing the quantitative distribution of microcystin and common potentially toxigenic cyanobacteria in US lakes and reservoirs. Harmful Algae.

[9] Wagner C., Adrian R. (2009). Cyanobacteria dominance: Quantifying the effects of climate change. Limnol. Oceanogr..

[10] Carey C.C., Ibelings B.W., Hoffmann E.P., Hamilton D.P., Brookes J.D. (2012). Eco-physiological adaptations that favour freshwater cyanobacteria in a changing climate. Water Res..

[11] Wilkinson A.A., Hondzo M., Guala M. (2018). Investigating abiotic drivers for vertical and temporal heterogeneities of cyanobacteria concentrations in lakes using a seasonal *in-situ* monitoring station. Water Resour. Res..

[12] Graham J.L., Loftin K.A., Meyer M.T., Ziegler A.C. (2010). Cyanotoxin Mixtures and Taste-and-Odor Compounds in Cyanobacterial Blooms from the Midwestern United States. Environ. Sci. Technol..

[13] Loftin K.A., Graham J.L., Hilborn E.D., Lehmann S.C., Meyer M.T., Dietze J.E., Griffith C.B. (2016). Cyanotoxins in inland lakes of the United States: Occurrence and potential recreational health risks in the EPA Natural Lakes Assessment 2007. Harmful Algae.

[14] Christoffersen K. (1996). Ecological implications of cyanobacterial toxins in aquatic food webs. Phycologia.

[15] Jacoby J.M., Collier D.C., Welch E.B., Hardy F.J., Crayton M. (2000). Environmental factors associated with a toxic bloom of Microcystis aeruginosa. Can. J. Fish. Aquat. Sci..

[16] Davis T.W., Berry D.L., Boyer G.L., Gobler C.J. (2009). The effects of temperature and nutrients on the growth and dynamics of toxic and non-toxic strains of *Microcystis* during cyanobacteria blooms. Harmful Algae.

[17] Hayes N.M., Vanni M.J. (2018). Microcystin concentrations can be predicted with phytoplankton biomass and watershed morphology. Inland Waters.

[18] Wilson A.E., Sarnelle O., Tillmanns A.R. (2006). Effects of cyanobacterial toxicity and morphology on the population growth of freshwater zooplankton: Meta-analyses of laboratory experiments. Limnol. Oceanogr..

[19] Urrutia-Cordero P., Ekvall M.K., Hansson L.A. (2015). Responses of cyanobacteria to herbivorous zooplankton across predator regimes: Who mows the bloom?. Freshw. Biol..

[20] Ger K.A., Otten T.G., DuMais R., Ignoffo T., Kimmerer W. (2018). In situ ingestion of Microcystis is negatively related to copepod abundance in the upper San Francisco Estuary. Limnol. Oceanogr..

[21] Triest L., Stiers I., Van Onsem S. (2016). Biomanipulation as a nature-based solution to reduce cyanobacterial blooms. Aquat. Ecol..

[22] Bierman V.J., Kaur J., DePinto J.V., Feist T.J., Dilks D.W. (2005). Modeling the role of zebra mussels in the proliferation of blue-green algae in Saginaw Bay, Lake Huron. J. Great Lakes Res..

[23] Fulton R.S., Paerl H.W. (1987). effects of colonial morphology on zooplankton utilization of algal resources during blue-green-algal (microcystis-aeruginosa) blooms. Limnol. Oceanogr..

[24] Demott W.R., Moxter F. (1991). Foraging on cyanobacteria by copepods—Responses to chemical defenses and resource abundance. Ecology.

[25] Yang Z., Kong F.X., Shi X.L., Zhang M., Xing P., Cao H.S. (2008). Changes in the morphology and polysaccharide content of Microcystis aeruginosa (Cyanobacteria) during flagellate grazing. J. Phycol..

[26] Yang Z., Kong F.X. (2012). Formation of large colonies: A defense mechanism of Microcystis aeruginosa under continuous grazing pressure by flagellate Ochromonas sp.. J. Limnol..

[27] Kim B.R., Nakano S., Kim B.H., Han M.S. (2006). Grazing and growth of the heterotrophic flagellate Diphylleia rotans on the cyanobacterium Microcystis aeruginosa. Aquat. Microb. Ecol..

[28] Mohamed Z., Alshehri A. (2013). Grazing on Microcystis aeruginosa and degradation of microcystins by the heterotrophic flagellate Diphylleia rotans. Ecotox. Environ. Saf..

[29] Van Wichelen J., van Gremberghe I., Vanormelingen P., Debeer A.E., Leporcq B., Menzel D., Codd G.A., Descy J.P., Vyverman W. (2010). Strong effects of amoebae grazing on the biomass and genetic structure of a Microcystis bloom (Cyanobacteria). Environ. Microbiol..

[30] Caron D.A., Countway P.D., Jones A.C., Kim D.Y., Schnetzer A. (2012). Marine protistan diversity. Ann. Rev. Mar. Sci..

[31] Domaizon I., Viboud S., Fontvieille D. (2003). Taxon-specific and seasonal variation in flagellates grazing on heterotrophic bacteria in the oligotrophic Lake Annecy – importance of mixotrophy. FEMS Microbiol. Ecol..

[32] Oikonomou A., Filker S., Breiner H., Stoeck T. (2015). Protistan diversity in a permanently stratified meromictic lake (Lake Alatsee, SW Germany). Environ. Microbial..

[33] Princiotta S.D., Sanders R.W. (2017). Heterotrophic and mixotrophic nanoflagellates in a mesotrophic lake: Abundance and grazing impacts across season and depth. Limnol Oceanogr..

[34] Wilken S., Huisman J., Naus-Wiezer S., Van Donk E. (2013). Mixotrophic organisms become more heterotrophic with rising temperature. Ecol. Lett..

[35] Princiotta S.D., Smith B.T., Sanders R.W. (2016). Temperature-dependent phagotrophy and phototrophy in a mixotrophic chrysophyte. J. Phycol..

[36] Jones H.L.J. (1997). A classification of mixotrophic protists based on their behavior. Freshw. Biol..

[37] Mitra A., Flynn K.J., Tillmann U., Raven J.A., Caron D., Stoecker D.K., Not F., Hansen P.J., Hallegraeff G., Sanders R.W. (2016). Defining planktonic protist functional groups on mechanisms for energy and nutrient acquisition: Incorporation of diverse mixotrophic strategies. Protist.

[38] Caron D.A., Sanders R.W., Lim E.L., Marrase C., Amaral L.A., Whitney S., Aoiki R.B., Porter K.G. (1993). Light-dependent phagotrophy in the freshwater mixotrophic chrysophyte Dinobryon cylindricum. Microb. Ecol..

[39] Wilken S., Verspagen J.M.H., Naus-Wiezer S., Van Donk E., Huisman J. (2014). Biological control of toxic cyanobacteria by mixotrophic predators: An experimental test of intraguild predation theory. Ecol. Appl..

[40] Holen D.A. (1999). Effects of prey abundance and light intensity on the mixotrophy chrysophyte Poterioochromonas malhamensis from a mesotrophic lake. Freshw. Biol..

[41] Zhang X., Hu H.Y., Hong Y., Yang J. (2008). Isolation of a Poterioochromonas capable of feeding on Microcystis aeruginosa and degrading microcystin-LR. FEMS Microbiol. Lett..

[42] Baek S.H., Hong S.S., Song S.Y., Lee H.O., Nakano S., Han M.S. (2009). Grazing Effects on Toxic and Non-Toxic Microcystis aeruginosa by the Mixotrophic Flagellate Ochromonas sp.. J. Freshw. Ecol..

[43] Van Donk E., Cerbin S., Wilken S., Helmsing N.R., Ptacnik R., Verschoor A.M. (2009). The effect of a mixotrophic chrysophyte on toxic and colony-forming cyanobacteria. Freshw. Biol..

[44] Wilken S., Wiezer S., Huisman J., Van Donk E. (2010). Microcystins do not provide anti-herbivore defense against mixotrophic flagellates. Aquat. Microb. Ecol..

[45] Sanders R.W., Porter K.G., Caron D.A. (1990). Relationship between phototrophy and phagotrophy in the mixotrophic chrysophyte Poterioochromonas malhamensis. Microb. Ecol..

[46] Holen D. (2001). The effects of heterotrophy on chlorophyll *a* and photosynthesis in a mixotrophic chrysophyte. Nova Hedwigia.

[47] Brett M.T., Kainz M.J., Taipale S.J., Seshan H. (2009). Phytoplankton, not allochthonous carbon, sustains herbivorous zooplankton production. Proc. Natl. Acad. Sci. USA.

[48] Hiltunen M., Honkanen M., Taipale S., Strandberg U., Kankaala P. (2017). Trophic upgrading via the microbial food web may link terrestrial dissolved organic matter to Daphnia. J. Plankton Res..

[49] Tranvik L., Porter K.G., Sieburth J.M. (1989). Occurance of bacterivory in Cryptomonas, a common freshwater phytoplankter. Oecologia.

[50] Urabe J., Gurung T.B., Yoshida T., Sekino T., Nakanishi M., Maruo M., Nakayama E. (2000). Diel changes in phagotrophy by Cryptomonas in Lake Biwa. Limnol. Oceanogr..

[51] B-Beres V., Grigorszky I., Vasas G., Borics G., Varbiro G., Nagy S.A., Borbely G., Bacsi I. (2012). The effects of Microcystis aeruginosa (cyanobacterium) on Cryptomonas ovata (Cryptophyta) in laboratory cultures: Why these organisms do not coexist in steady-state assemblages?. Hydrobiologia.

[52] Grujcic V., Nuy J.K., Salcher M.M., Shabarova T., Kasalicky V., Boenigk J., Jensen M., Simek K. (2018). Cryptophyta as major bacterivores in freshwater summer plankton. ISME J..

[53] Worm J., Sondergaard M. (1998). Dynamics of heterotrophic bacteria attached to Microcystis spp. (Cyanobacteria). Aquat. Microb. Ecol..

[54] Seymour J.R., Amin S.A., Raina J.B., Stocker R. (2017). Zooming in on the phycosphere: The ecological interface for phytoplankton-bacteria relationships. Nat. Microbiol..

[55] Kamjunke N., Boing W., Voigt H. (1997). Bacterial and primary production under hypertrophic conditions. Aquat. Microb. Ecol..

[56] Kirkwood A.E., Nalewajko C., Fulthorpe R.R. (2006). The effects of cyanobacterial exudates on bacterial growth and biodegradation of organic contaminants. Microb. Ecol..

[57] Shen H., Niu Y., Xie P., Tao M., Yang Xi. (2011). Morphological and physiological changes in Microcystis aeruginosa as a result of interactions with heterotrophic bacteria. Freshw. Biol..

[58] Monchamp M.E., Pick F.R., Beisner B.E., Maranger R. (2014). Nitrogen Forms Influence Microcystin Concentration and Composition via Changes in Cyanobacterial Community Structure. PLoS ONE.

[59] Fu Y., O’Kelly C.O., Sieracki M., Distel D.L. (2003). Protistan grazing analysis by flow cytometry using prey labeled by in vivo expression of fluorescent proteins. Appl. Environ. Microbiol..

[60] Gonzalez J.M., Sherr E.B., Sherr B.F. (1993). Differential feeding by marine flagellates on growing versus starving, and on motile versus nonmotile, bacterial prey. Mar. Ecol. Prog. Ser..

[61] Ou D., Song L., Gan N., Chen W. (2005). Effects of microcystins on and toxin degradation by *Poterioochromonas* sp.. Environ. Toxicol..

[62] Reiter M.A. (1997). A simple fluorescent labeling technique for the marking of algae in mixed assemblages. J. Freshw. Ecol..

[63] Sherr B.F., Sherr E.B., Fallon R.D. (1987). Use of monodispersed, fluorescently labeled bacteria to estimate in-situ protozoan bacterivory. Appl. Environ. Microbiol..

[64] Shoemaker J., Tettenhorst D., Delacruz A. (2015). Method 544: Determination of Microcystins and Nodularin in Drinking Water by Solid Phase Extraction and Liquid Chromatography/Tandem Mass Spectrometry (LC/MS/MS).

[65] Hothorn T., Bretz F., Westfall P. (2008). Simultaneous interference in general parametric models. Biom. J..

