# Supplementary material for: Production of Cyanotoxins by Microcystis aeruginosa Mediates Interactions with the Mixotrophic Flagellate Cryptomonas
Source: Toxins (Basel). 2019 Apr 15;11(4):223. doi: 10.3390/toxins11040223 (PMC6520739; doi:10.3390/toxins11040223)
Supplement: Supplementary file 1 [file toxins-11-00223-s001.pdf]

Supplementary Materials: Production of Cyanotoxins by *Microcystis aeruginosa* Mediates Interactions with The Mixotrophic Flagellate *Cryptomonas*

Sarah DeVaul Princiotta, Susan P. Hendricks and David S. White

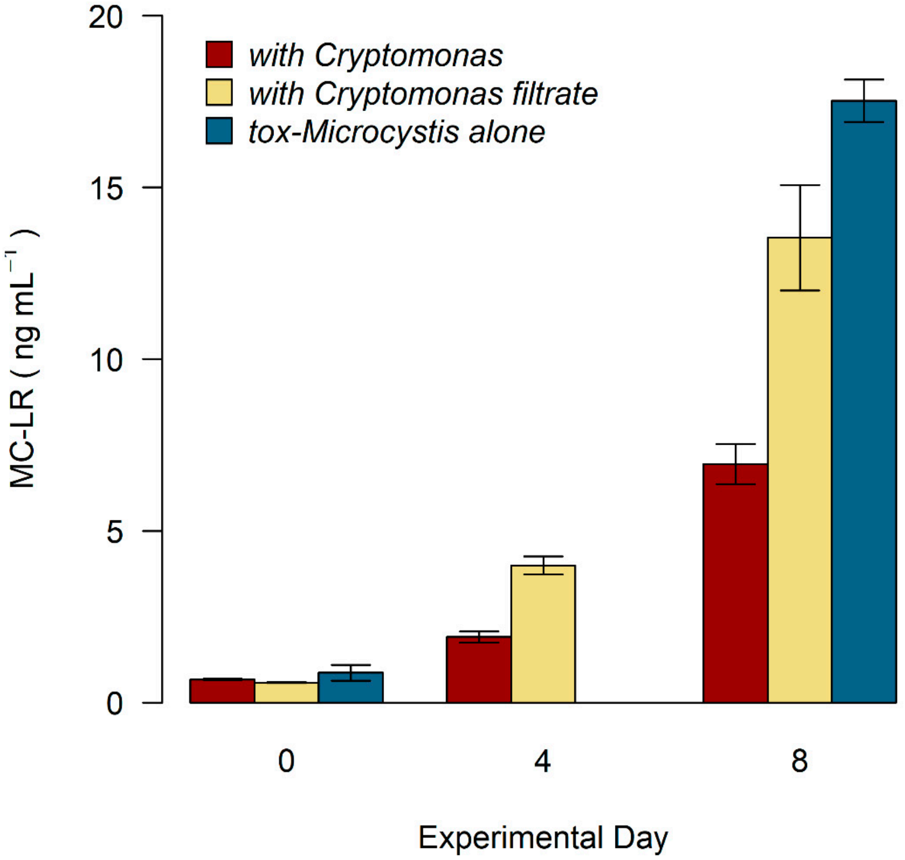

Figure S1. Concentration of microcystin-LR (MC-LR) without correction for cell abundance, as measured in tox-*Microcystis* after incubation with *Cryptomonas*, filtrate from the mixotroph, or alone in monoculture. Note that MC-LR was not measured in tox-*Microcystis* monoculture on day 4.

Table S1. Average dissolved organic carbon (DOC) derived from unialgal cultures, measured in triplicate samples.

**Species**	**DOC (mg L^{-1})**	**Std. Error**
Cryptomonas sp.	19.72	0.87
tox-*Microcystis*	5.38	0.47
nt-*Microcystis*	6.27	0.17
